# Supplementary material for: Modeling glioblastoma heterogeneity as a dynamic network of cell states
Source: Mol Syst Biol. 2021 Sep 16;17(9):e10105. doi: 10.15252/msb.202010105 (PMC8444284; doi:10.15252/msb.202010105)
Supplement: Supplementary file 6 — Source Data for Figure 5 [file MSB-17-e10105-s004.zip › Figure5A_sourcedata/GSEA_3071/hallmarks_stateII.GseaPreranked.1623742536692/HALLMARK_UV_RESPONSE_DN.html]

Details for gene set HALLMARK\_UV\_RESPONSE\_DN[GSEA]

|  || Dataset | state23071 |
| Phenotype | NoPhenotypeAvailable |
| Upregulated in class | na\_pos |
| GeneSet | HALLMARK\_UV\_RESPONSE\_DN |
| Enrichment Score (ES) | 0.22540687 |
| Normalized Enrichment Score (NES) | 1.2386636 |
| Nominal p-value | 0.16666667 |
| FDR q-value | 0.27286822 |
| FWER p-Value | 0.972 |
Table: GSEA Results Summary

  

Fig 1: Enrichment plot: HALLMARK\_UV\_RESPONSE\_DN      
 Profile of the Running ES Score & Positions of GeneSet Members on the Rank Ordered List

  

| PROBE | GENE SYMBOL | GENE\_TITLE | RANK IN GENE LIST | RANK METRIC SCORE | RUNNING ES | CORE ENRICHMENT || 1 | F3 |  |  | 13 | 1.555 | 0.0369 | Yes |
| 2 | MT1E |  |  | 106 | 1.033 | 0.0408 | Yes |
| 3 | CAV1 |  |  | 134 | 0.988 | 0.0596 | Yes |
| 4 | EFEMP1 |  |  | 165 | 0.932 | 0.0762 | Yes |
| 5 | MET |  |  | 183 | 0.897 | 0.0951 | Yes |
| 6 | DLC1 |  |  | 208 | 0.868 | 0.1116 | Yes |
| 7 | MGLL |  |  | 227 | 0.848 | 0.1290 | Yes |
| 8 | COL1A1 |  |  | 294 | 0.761 | 0.1323 | Yes |
| 9 | SERPINE1 |  |  | 299 | 0.756 | 0.1508 | Yes |
| 10 | LPAR1 |  |  | 421 | 0.668 | 0.1381 | Yes |
| 11 | SLC7A1 |  |  | 448 | 0.651 | 0.1484 | Yes |
| 12 | DAB2 |  |  | 491 | 0.631 | 0.1543 | Yes |
| 13 | PDLIM5 |  |  | 526 | 0.613 | 0.1617 | Yes |
| 14 | FHL2 |  |  | 541 | 0.607 | 0.1739 | Yes |
| 15 | KCNMA1 |  |  | 570 | 0.590 | 0.1822 | Yes |
| 16 | DUSP1 |  |  | 599 | 0.578 | 0.1902 | Yes |
| 17 | ID1 |  |  | 769 | 0.509 | 0.1614 | Yes |
| 18 | MYC |  |  | 790 | 0.502 | 0.1694 | Yes |
| 19 | SYNJ2 |  |  | 799 | 0.498 | 0.1803 | Yes |
| 20 | ANXA2 |  |  | 839 | 0.488 | 0.1832 | Yes |
| 21 | ADORA2B |  |  | 841 | 0.486 | 0.1955 | Yes |
| 22 | SNAI2 |  |  | 842 | 0.486 | 0.2081 | Yes |
| 23 | RND3 |  |  | 860 | 0.482 | 0.2163 | Yes |
| 24 | NFKB1 |  |  | 874 | 0.478 | 0.2254 | Yes |
| 25 | DDAH1 |  |  | 1028 | 0.434 | 0.1987 | No |
| 26 | COL11A1 |  |  | 1163 | 0.404 | 0.1758 | No |
| 27 | ITGB3 |  |  | 1325 | 0.374 | 0.1456 | No |
| 28 | COL5A2 |  |  | 1362 | 0.366 | 0.1461 | No |
| 29 | NRP1 |  |  | 1372 | 0.362 | 0.1532 | No |
| 30 | NR3C1 |  |  | 1510 | 0.339 | 0.1280 | No |
| 31 | FZD2 |  |  | 1545 | 0.332 | 0.1281 | No |
| 32 | TENT4A |  |  | 1593 | 0.325 | 0.1248 | No |
| 33 | PIK3CD |  |  | 1689 | 0.312 | 0.1093 | No |
| 34 | ATP2B4 |  |  | 1713 | 0.308 | 0.1116 | No |
| 35 | NEK7 |  |  | 2048 | 0.267 | 0.0356 | No |
| 36 | LDLR |  |  | 2128 | 0.260 | 0.0227 | No |
| 37 | KALRN |  |  | 2182 | 0.254 | 0.0161 | No |
| 38 | RBPMS |  |  | 2261 | -0.255 | 0.0034 | No |
| 39 | GJA1 |  |  | 2366 | -0.266 | -0.0156 | No |
| 40 | CAP2 |  |  | 2406 | -0.272 | -0.0182 | No |
| 41 | CDON |  |  | 2454 | -0.278 | -0.0227 | No |
| 42 | SIPA1L1 |  |  | 2547 | -0.293 | -0.0380 | No |
| 43 | PRKAR2B |  |  | 2550 | -0.293 | -0.0309 | No |
| 44 | SDC2 |  |  | 2574 | -0.297 | -0.0289 | No |
| 45 | IGF1R |  |  | 2700 | -0.316 | -0.0518 | No |
| 46 | PRKCA |  |  | 2757 | -0.327 | -0.0572 | No |
| 47 | BMPR1A |  |  | 2800 | -0.335 | -0.0590 | No |
| 48 | IGFBP5 |  |  | 2820 | -0.339 | -0.0550 | No |
| 49 | NR1D2 |  |  | 2877 | -0.350 | -0.0598 | No |
| 50 | ATRN |  |  | 2908 | -0.357 | -0.0580 | No |
| 51 | PTEN |  |  | 2929 | -0.361 | -0.0537 | No |
| 52 | APBB2 |  |  | 3023 | -0.378 | -0.0670 | No |
| 53 | PIK3R3 |  |  | 3151 | -0.414 | -0.0878 | No |
| 54 | AMPH |  |  | 3226 | -0.435 | -0.0949 | No |
| 55 | FYN |  |  | 3262 | -0.444 | -0.0921 | No |
| 56 | ARHGEF9 |  |  | 3270 | -0.446 | -0.0824 | No |
| 57 | ATRX |  |  | 3509 | -0.546 | -0.1273 | No |
| 58 | ATXN1 |  |  | 3586 | -0.598 | -0.1307 | No |
| 59 | MAGI2 |  |  | 3695 | -0.681 | -0.1399 | No |
| 60 | HAS2 |  |  | 3724 | -0.702 | -0.1287 | No |
| 61 | INPP4B |  |  | 3760 | -0.742 | -0.1183 | No |
| 62 | CDKN1B |  |  | 3812 | -0.800 | -0.1103 | No |
| 63 | SRI |  |  | 3885 | -0.909 | -0.1046 | No |
| 64 | PLPP3 |  |  | 3900 | -0.946 | -0.0837 | No |
| 65 | MMP16 |  |  | 3983 | -1.171 | -0.0738 | No |
| 66 | COL1A2 |  |  | 3988 | -1.187 | -0.0441 | No |
| 67 | NFIB |  |  | 4007 | -1.310 | -0.0148 | No |
| 68 | CELF2 |  |  | 4027 | -1.437 | 0.0176 | No |
Table: GSEA details [plain text format]

  

Fig 2: HALLMARK\_UV\_RESPONSE\_DN: Random ES distribution      
 Gene set null distribution of ES for **HALLMARK\_UV\_RESPONSE\_DN**
